# Shape‐Encoded Hydrogel Sensor Particles Enable Multiplex Odorant Detection Through Deep‐learning Classification

**DOI:** 10.1002/smll.202507903

**Published:** 2025-10-22

**Authors:** Sho Takamori, Taisei Kawakami, Tomoko Ohnishi, Hisatoshi Mimura, Toshihisa Osaki, Norihisa Miki, Shoji Takeuchi

**Affiliations:** ^1^ Artificial Cell Membrane Systems Group Kanagawa Institute of Industrial Science and Technology 3‐2‐1 Sakado, Takatsu‐ku Kawasaki Kanagawa 213‐0012 Japan; ^2^ Department of Mechanical Engineering Faculty of Science and Technology Keio University 3‐14‐1 Hiyoshi, Kohoku‐ku Yokohama Kanagawa 223–8522 Japan; ^3^ Institute of Industrial Science The University of Tokyo 4‐6‐1 Komaba, Meguro‐ku Tokyo 153–8505 Japan; ^4^ Department of Mechano‐Informatics Graduate School of Information Science and Technology The University of Tokyo 7‐3‐1 Hongo, Bunkyo‐ku Tokyo 113–8656 Japan

**Keywords:** convolutional neural network, hydrogel encoding, hydrogel particles, odorant sensor cells, shape classification

## Abstract

Simultaneous detection of multiple odorants is a major challenge in the development of portable, cell‐based biohybrid sensors, primarily due to the difficulty of distinguishing between different sensor cell types. Here, a strategy that encodes odorant sensor cell types using the shape of hydrogel particles, enabling shape‐based identification through deep learning is reported. Each particle shape corresponds to a unique sensor cell type expressing a distinct odorant receptor (OR). A convolutional neural network is trained to classify these shapes with high accuracy, and the resulting shape identification scheme is applied to time‐lapse fluorescence images of mixed particles exposed to single odorants. This enabled reliable assignment of particle identity and extraction of shape‐specific fluorescence signals. Distinct odorant‐dependent responses are observed, consistent with the known ligand specificities of the corresponding ORs. While this study focuses on individual odorants, the shape‐based approach provides a position‐independent, scalable method for multiplexed odorant detection. This framework supports the development of compact, high‐throughput biohybrid sensors for safety, environmental monitoring, and diagnostic applications.

## Graphical abstract

Odorant sensor cells expressing odorant receptors, odorant co‐receptors, and fluorescent calcium indicators are promising for multiplex odorant detection. This study proposes a platform for simultaneous fluorescence analysis of shape‐encoded hydrogel particles containing these cells. By encoding cell types through particle shape and identifying shapes with a trained deep‐learning model, sensor particles can be distinguished without position‐based labeling.

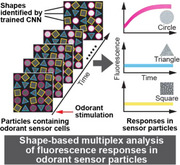

## 1 Introduction

Odor detection is essential for safety, environmental monitoring, and diagnostics. Conventional technologies, such as trained animals or humans,^[^ ^1^, ^2^ ^]^ gas sensors,^[^ ^3^ ^]^ and gas chromatography‐mass spectrometry (GC‐MS),^[^ ^4^ ^]^ offer high sensitivity but rely on bulky instruments, limiting portability and field use. Biohybrid odorant sensors^[^ ^5^, ^6^, ^7^, ^8^, ^9^, ^10^, ^11^, ^12^, ^13^ ^]^ have emerged as promising alternatives. These systems typically use cells engineered to express odorant receptors (ORs) and co‐receptors (Orcos), combined with calcium‐sensitive fluorescent indicators such as GCaMP,^[^ ^12^, ^14^, ^15^ ^]^ to enable selective, sensitive, and potentially portable odor detection. Many studies have already demonstrated OR‐based biosensors that detect individual odorants using a single type of OR‐expressing sensor cell.^[^ ^6^, ^11^, ^12^, ^13^, ^16^, ^17^ ^]^ To extend sensing capabilities, researchers have attempted to increase the number of distinct sensor cells. A widely adopted approach involves spatially patterning different sensor cells into arrays on 2D substrates,^[^ ^15^, ^18^, ^19^ ^]^ enabling interpretation of responses based on their known positions. While this array‐based approach is effective, its complexity increases as the number of cell types grows, which may limit scalability. As an alternative, confining sensor cells within hydrogel particles using various encoding strategies^[^ ^20^, ^21^, ^22^ ^]^ enables unique labeling without the need for spatial patterning. Moreover, recent advances in open‐source deep learning frameworks and libraries have made high‐throughput image‐based analysis of such particles increasingly accessible.

In this study, we present a method to distinguish sensor cell types using deep learning‐based image analysis, without the need for spatial patterning. We embed different odorant sensor cells in hydrogel particles, each fabricated in a unique shape corresponding to a specific cell type. We then train a convolutional neural network (CNN) to classify particle shapes and implement shape identification. By identifying shape‐encoded sensor particles in randomly mixed suspensions, our method eliminates the need for spatially patterned arrays and therefore remains straightforward even as the number of sensor cell types increases, enabling simultaneous multiplex sensing. To evaluate the method, we add individual odorants to mixtures of shape‐encoded particles and analyze fluorescence responses based on the assigned shapes. While this study focuses on single‐odorant inputs, the proposed shape‐based strategy offers a position‐independent and scalable solution for multiplexed sensor readout, providing a foundation for compact, portable biohybrid odor sensing platforms.

## 2 Results

### 2.1 Formation of Hydrogel Particles Containing Odorant Sensor Cells

**Figure 1:**
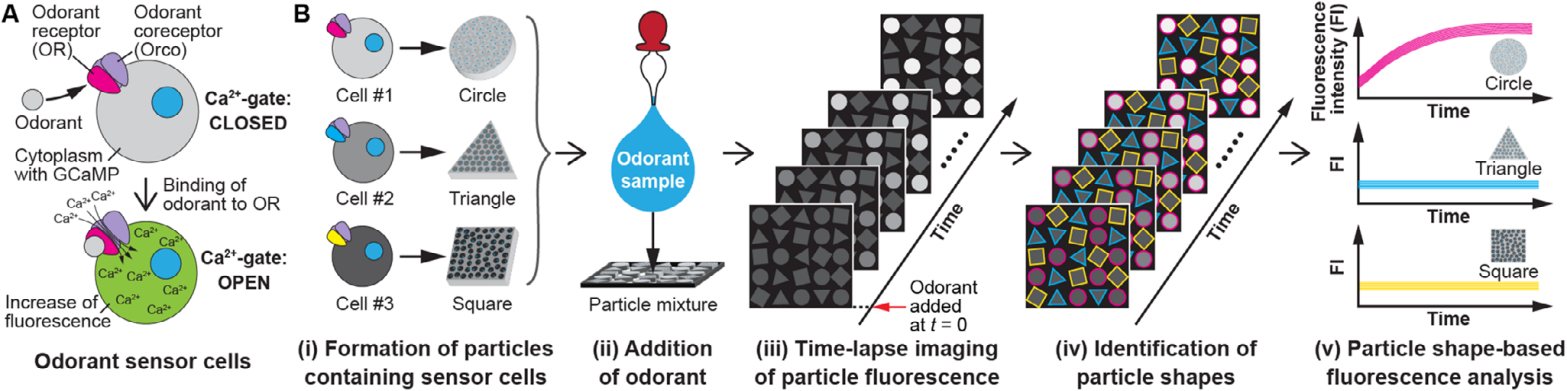
Shape‐encoded hydrogel particles for multiplexed analysis of odorant sensor cells. A) Odorant sensor cells are engineered to express a specific OR, along with Orco and a calcium‐sensitive fluorescent indicator, enabling them to emit fluorescence in response to odorant‐induced calcium influx. B) Workflow of the proposed method: i) different types of sensor cells, each expressing a distinct OR, are embedded in hydrogel particles with shape‐specific geometries; ii) an odorant sample is added to the mixed particle suspension; iii) time‐lapse imaging of particle fluorescence is performed following odorant addition; iv) particle shapes are identified by deep learning‐based image analysis; and v) fluorescence responses are analyzed according to the identified particle shapes.

**Figure 2:**
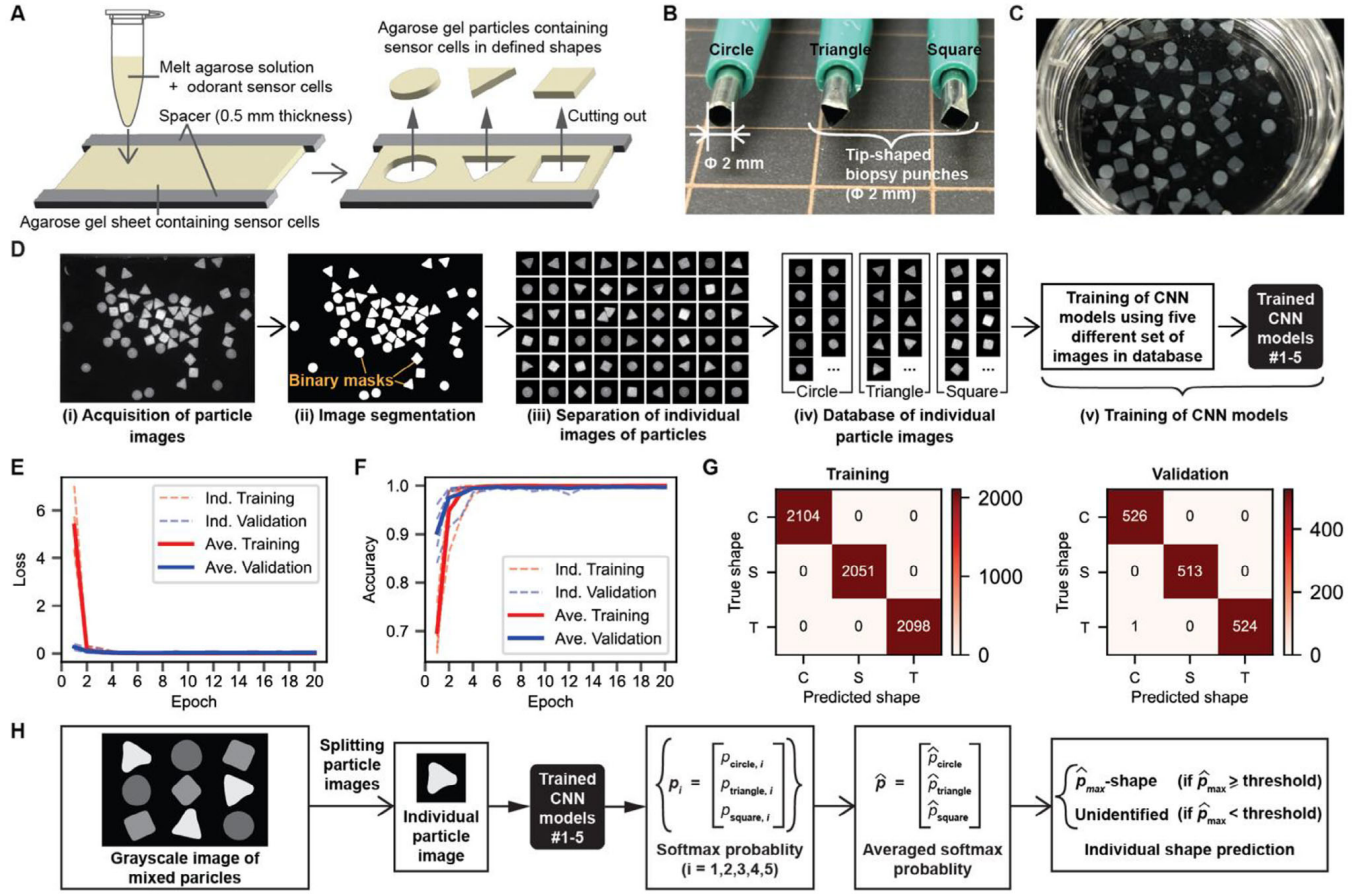
Development of the particle shape identification scheme. A) Fabrication of agarose gel particles containing odorant sensor cells in three distinct shapes. B) Biopsy punches with custom‐deformed tips used for hydrogel molding. C) Representative images of cell‐containing agarose particles molded into circle, triangle, and square shapes. D) Schematic workflow from acquisition of training particle images to training of CNN models. E) Categorical cross‐entropy loss during CNN model training and validation. F) Training and validation accuracy of the CNN models. G) Aggregated confusion matrices for training (left) and validation (right) across five independently trained CNN models. Shape categories: circle (“C”), square (“S”), and triangle (“T”). H) Shape identification scheme using ensemble predictions from five CNN models for classifying the shapes of individual sensor cell‐containing particles.

To prepare odorant sensors for shape‐based simultaneous response analysis (**Figure** **1**), we produced hydrogel particles containing odorant sensor cells (see Section 4, Experimental Section for details). Briefly, sensor cells were harvested and dispersed into melted 2% agarose solution (gel point ≈20 °C), which was then spread between two silicone spacers using a glass microscope slide (thickness: 0.5 mm). After cooling the agarose to 4 °C for 30 min to induce gelation, the resulting gel sheet containing cells was manually cut into particles using biopsy punches. The punch tips were reshaped to form one of three geometries (circle, triangle, or square) depending on the cell type (**Figure** **2**A,B). As shown in Figure 2C, this method reliably produced sensor cell‐containing hydrogel particles with predetermined shapes.

### 2.2 Development of Particle Shape Identification Scheme

To enable automatic identification of hydrogel particle shapes containing odorant sensor cells, we developed a deep learning‐based shape identification scheme.

We first prepared a training dataset (Figure 2D; see Section 4, Experimental Section for details) containing example images (correct answers) of cell‐containing hydrogels in three different shapes. Briefly, grayscale fluorescence images of sensor cell‐containing hydrogel particles in three shapes (circle, triangle, square) were segmented using *Cellpose 2.0* ^[^ ^23^, ^24^ ^]^ to generate binary masks. We chose *Cellpose 2.0* for segmentation because it allows users to create a user‐trained model optimized for segmenting cell‐like objects in micrographs whose characteristics vary depending on the microscopes used and sample conditions. Identified particle segments were filtered by pixel size to remove unwanted broken or overlapping objects. Grayscale regions corresponding to the particles were extracted using the segmentation masks via Boolean operations. The extracted pixels corresponding to the particles in grayscale images were centered in 128 × 128 pixel zero‐padded images without resizing. The resulting individual particle images were manually sorted into different folders (named as circle, triangle, square) by identifying shapes, yielding ≈2600 images per shape.

We then trained a CNN using a basic sequential architecture (Figure 2D; see Section 4, Experimental Section for details). Five independent CNN models were trained on this dataset of particle images, using independent 80:20 training‐validation splits and categorical cross‐entropy as the loss function. As shown in Figure 2E, the loss converged rapidly within 2–6 epochs for both training and validation sets, with corresponding accuracies approaching 100% in Figure 2F. Confusion matrices summarizing training and validation results confirmed high classification accuracy, with 100% performance on the training set and a misclassification rate of only 0.06% on the validation set (Figure 2G).

Finally, we performed shape identification following a developed scheme using the five trained CNN classifiers (Figure 2H; see Section 4, Experimental Section for details). For each segmented grayscale particle image, softmax probability outputs from the five models were averaged. If the highest softmax probability value exceeded a predefined threshold (0.5; see Section 4, Experimental Section for details), a shape corresponding to the highest softmax probability was assigned following the standard argmax‐based classification procedure; otherwise, the particle was labeled as “unidentified.”

These results demonstrate that our CNN models achieved high classification accuracy without overfitting and that the ensemble‐based identification scheme provides a robust framework for automatic shape recognition.

### 2.3 Particle Shape Identification Using Test Images

**Figure 3:**
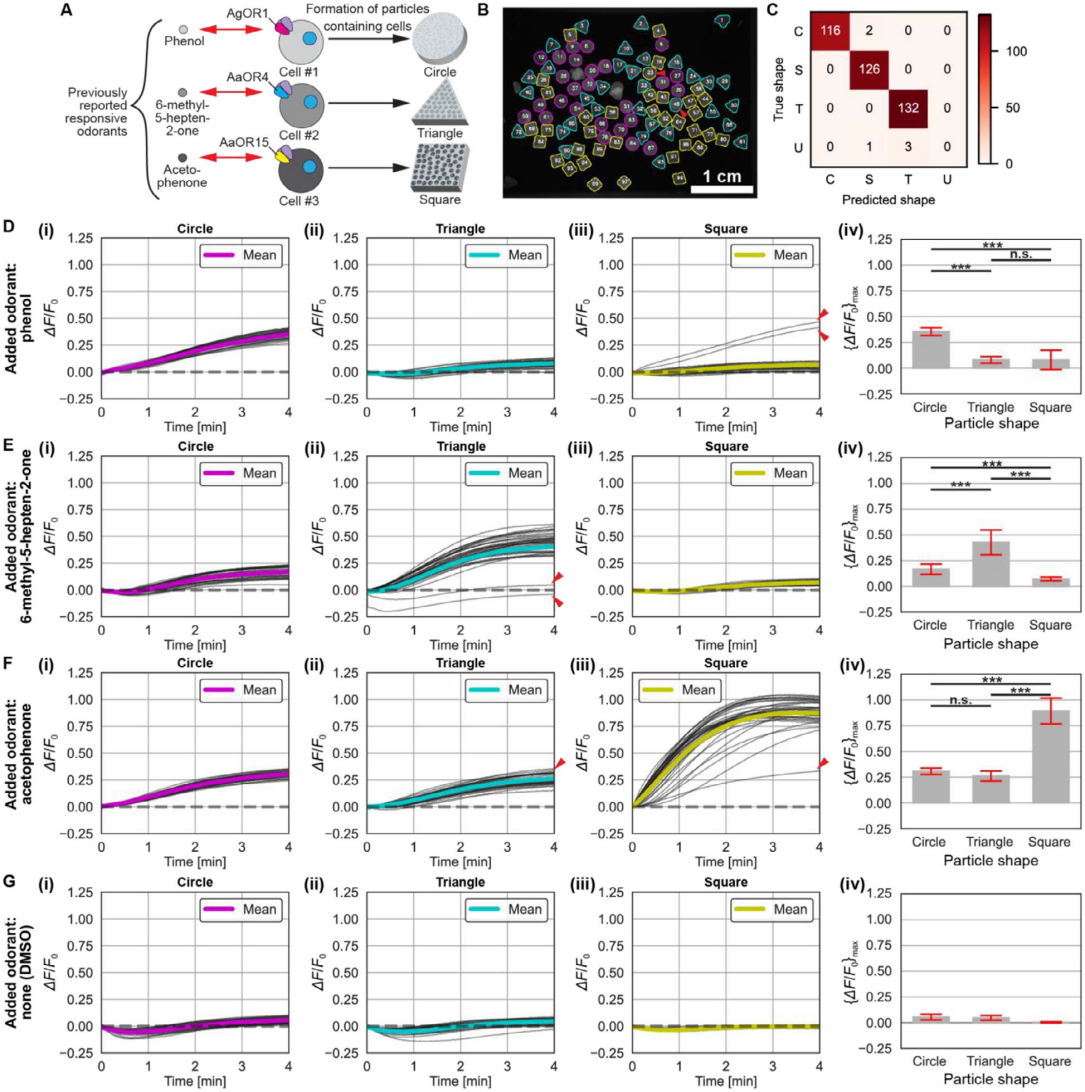
Shape‐based multiplexed analysis of odorant sensor particles. A) Schematic illustration of three types of odorant sensor cells, each expressing an OR previously reported to respond strongly to a specific odorant, embedded in agarose particles of distinct shapes. B) Representative fluorescence image of sensor cell‐containing particles, with classified shapes highlighted in color (circle: magenta; triangle: cyan; square: yellow). Odorant added: acetophenone. Numbers indicate unique particle indices assigned during shape identification. Red arrowheads mark particles with shape misidentification. C) Aggregated confusion matrix summarizing shape identification results across three odorant test conditions and one DMSO control. Shape labels: circle (“C”), triangle (“T”), square (“S”), and unidentified (“U”). (D‐G) Time‐course plots of fluorescence change (*ΔF*/*F*₀) for each identified shape following single‐odorant addition i–iii), and corresponding bar plots of peak responses iv): phenol D), 6‐methyl‐5‐hepten‐2‐one E), acetophenone F), and DMSO control G). Red arrowheads in plots indicate particles misidentified by shape. Gray bars indicate mean values; red error bars represent standard deviation. Number of particles (*n*_C_, *n*_T_, *n*_S_): (31, 30, 35) in D; (29, 40, 32) in E; (26, 33, 36) in F; (30, 32, 26) in G. See 4. Experimental Section for details of statistical analysis and *p*‐value annotations.

To evaluate the performance of the established shape identification scheme (Figure 2H), we applied it to newly acquired test images. We conducted time‐lapse measurements in which one of three odor molecules or a negative control solution containing DMSO used for dissolving odor molecules was added at *t* = 0 min to a mixture of agarose gel particles, each containing different odorant sensor cells (see Section 4, Experimental Section for details). Each particle shape corresponded to a specific sensor cell type expressing an OR that was previously reported significantly responsive to a target odorant: AgOR1^[^ ^25^ ^]^ for phenol, AaOR4^[^ ^26^ ^]^ (AaegOR4) for 6‐methyl‐5‐hepten‐2‐one, and AaOR15^[^ ^27^ ^]^ (AaegOR15) for acetophenone (**Figure** **3**A). Similarly to the preparation of the training dataset, particles were segmented with *Cellpose 2.0*,^[^ ^23^, ^24^ ^]^ individual particle regions in grayscale images were extracted, the extracted grayscale pixels corresponding to individual particles were saved as 128 × 128 pixel zero‐padded images without resizing, and identification of individual particle shapes was conducted using these images and the established shape identification scheme (Figure 2H). A representative result in Figure 3B shows high accuracy of shape identification, with a misidentification rate of ≈2.1% (2/96) (see Figure S1, Supporting Information for other results). Across all four experiments, the overall error rate was 1.6% (6/380). In all cases, errors were attributed to visually ambiguous particles. The aggregated confusion matrix further confirmed the high identification accuracy on test images (Figure 3C). These results indicate that the developed scheme can reliably identify hydrogel particle shapes with minimal classification error.

### 2.4 Shape‐Based Simultaneous Response Analysis

To demonstrate shape‐based simultaneous response analysis of sensor particles, we analyzed time‐lapse images from the test odorant stimulation experiments based on the identified shapes of the agarose gel particles. For each particle, we computed the normalized fluorescence change defined as *ΔF*/*F*_0_ = (*F*(*t*) – *F*_0_)/ *F*_0_, where *F*(*t*) is the mean fluorescence intensity of individual particles at time *t* and *F*_0_ (= *F*(0)) is the initial mean intensity of the particles at *t* = 0 min.

First, phenol induced a clear response in circle‐shaped particles, with *ΔF*/*F*_0_ exceeding 0.3 within 4 min, while triangle and square particles showed only minor increases (*ΔF*/*F*_0_ < 0.1) (Figure 3D (i–iii); see Figure S4A, Supporting Information for before and after images). Bar plots of the maximum *ΔF*/*F*_0_ (Figure 3D (iv)) confirmed significantly stronger responses in circle particles. Next, 6‐methyl‐5‐hepten‐2‐one triggered the strongest response in triangle particles (*ΔF*/*F*_0_ > 0.3 within 3 min), while responses in circle and square particles remained lower (*ΔF*/*F*_0_ < 0.2) (Figure 3E (i–iv); see Figure S4B, Supporting Information for before and after images). Third, acetophenone induced the highest response in square particles, reaching *ΔF*/*F*_0_ ≈0.8 within 3 min. Circle and triangle particles showed moderate responses (*ΔF*/*F*_0_ > 0.25) within 3–4 min (Figure 3F(i–iv); see Figure S4C, Supporting Information for before and after images). Finally, the DMSO control induced no significant changes in *ΔF*/*F*_0_ (Figure 3G; see Figure S4D, Supporting Information for before and after images), confirming the odorant‐triggered nature of the responses. A small number of misidentified particles, marked by red arrowheads in Figure 3D–F, were observed and left included in the analysis, accounting for 2/96 (≈2.1%), 2/101 (≈2.0%), 2/95 (≈2.1%) of the analyzed particles in the odorant stimulation experiments using phenol, 6‐methyl‐5‐hepten‐2‐one, and acetophenone respectively.

These results indicate that: 1) circle particles contain sensor cells with the highest response to phenol (cells expressing AgOR1), 2) triangle particles to 6‐methyl‐5‐hepten‐2‐one (cells expressing AaOR4), and 3) square particles to acetophenone (cells expressing AaOR15) (Figure 3A).

## 3 Discussion

In this study, we developed a method for simultaneously analyzing the responses of cell‐based odorant sensor particles by identifying particle shapes and analyzing their fluorescence (Figure 1B). We first generated hydrogel particles containing odorant sensor cells in three distinctive shapes (Figure 2C), sorted their images by shape, and created an image database (Figure 2D). Using this dataset, we independently trained five CNN models (Figure 2D–G) and constructed a particle shape identification scheme based on their predictions (Figure 2H). When applied to newly acquired test images, this scheme achieved high classification accuracy, confirming its robustness (Figure 3B,C). Finally, based on the identified shapes, we analyzed fluorescence responses from individual particles (Figure 3D–G).

We embedded sensor cells at high density (≈10^7^–10^8^ cells mL^−1^) within particles to smooth fluorescence responses at the particle level, as individual cells exhibited substantial variability in baseline fluorescence and odorant‐induced responses (Figure S2, Supporting Information). Given the particle thickness of ≈0.5 mm, which approaches the diffusion limit for small molecules in hydrogels, dense packing likely increased the likelihood that responsive cells were located near the surface, where odorants could diffuse more readily and trigger faster responses. Nevertheless, we observed variability in fluorescence signals even among particles of the same shape (Figure 3D–G), which we attribute mainly to uneven odorant distribution. In our setup, odorants were manually added at six locations per well to reach a final concentration of 100 µM, but the addition process took ≈7 s (Figure S5, Supporting Information), and full diffusion likely took longer. These transient concentration gradients may have contributed to differences in response timing across particles. Optimizing the odorant delivery method could help reduce this variability. In addition, we noticed a slight uneven thickness of the hydrogel sheet before making particles using punches, which is likely due to manual spreading of cell‐dispersed agarose solution using the sharp edges of glass slides between silicone spacers. To prevent the occurrence of such uneven thickness, another strategy such, as molding in the 3D confinement, could be investigated.

We used geometric shapes as a form of hydrogel encoding^[^ ^20^, ^21^, ^22^ ^]^ to label sensor cell types within hydrogel particles, owing to several practical advantages. First, shape‐defined particles could be quickly fabricated using biopsy punches with manually reshaped tips (Figure 2B), thereby avoiding the need for more complex fabrication techniques such as molding^[^ ^28^, ^29^, ^30^, ^31^, ^32^ ^]^ or lithography.^[^ ^33^, ^34^, ^35^ ^]^ Second, the resulting shapes were visually distinguishable, enabling efficient manual sorting of particle images for database construction (Figure 2D) without requiring additional labeling or annotation. These advantages facilitated the rapid development of our complete analysis scheme. We consider this shape‐based strategy to be theoretically scalable, assuming reliable fabrication of distinct geometries. However, more advanced fabrication techniques may become necessary for producing highly complex shapes. Alternative encoding strategies, such as graphical patterns,^[^ ^36^, ^37^ ^]^ may be applicable to similar systems but typically require systematic decoding methods, including automated annotation.^[^ ^38^ ^]^ Similarly, color‐based encoding^[^ ^39^, ^40^ ^]^ may face limitations in available staining options as the number of target sensor types increases. Therefore, the choice of an appropriate encoding strategy should be guided by the scalability and application‐specific requirements of the system.

While the validation dataset yielded very high classification accuracy (1/1564 ≈0.06%; Figure 2G), the test dataset showed a slightly higher misidentification rate (6/380 ≈1.6%; Figure 3C). The slightly lower accuracy in the test set likely resulted from overlapping or aggregated particles, which were not present in the cleaner training set (Figure 2D(iv)). Unlike the clean training dataset, the test images often included partially overlapping or aggregated particles that were difficult to segment accurately and could not be reliably excluded using area‐based thresholding (Figure 3B). Indeed, all misclassifications in Figure 3B occurred in such problematic cases, which ideally should have been labeled as “unidentified” but were instead misclassified as square or triangle shapes. Nonetheless, the overall error rate remained low and appeared to have minimal impact on the fluorescence response analyses.

The ORs used in this study have been previously characterized as exhibiting strong responses to their respective odorants: AgOR1 to phenol,^[^ ^25^ ^]^ AaOR4 to 6‐methyl‐5‐hepten‐2‐one,^[^ ^26^ ^]^ and AaOR15 to acetophenone.^[^ ^27^ ^]^ In agreement with these reports, our shape‐encoded sensor particles showed the strongest fluorescence responses in the anticipated OR‐odorant combinations (Figure 3D (i,iv),E (ii,iv),F (iii, iv)). We confirmed the reproducibility of these responses in another experiment shown in Figures S6 and S7 and also in our recent study reporting 2D arrays of smaller cell‐containing hydrogel particles.^[^ ^42^ ^]^ Interestingly, moderate responses were also detected from non‐corresponding ORs (Figure 3E (i),F(i,ii)), possibly attributable to the known cross‐reactivity and broad ligand tuning observed in insect ORs,^[^ ^41^ ^]^ despite the limited apparent structural similarity among the three odorants. These results highlight the value of pattern‐based decoding across multiple ORs, rather than relying on highly selective single‐receptor responses, for robust odor discrimination. Although this study examined a limited set of three ORs and three odorants, expanding the molecular and receptor diversity could enable more subtle pattern recognition and facilitate the multiplexed detection of complex odorant mixtures. Moreover, increasing receptor diversity could help mitigate the risk that mixtures of odorants produce response patterns resembling those of single odorants or other odorant combinations. When more than two odorants are presented simultaneously, the resulting patterns may become even more complex, as recently demonstrated with a three‐odorant mixture in a separate study,^[^ ^42^ ^]^ further underscoring the importance of employing diverse receptor repertoires for reliable discrimination.

One strength of our approach is that automated analysis can greatly improve the efficiency of odorant detection. Currently, experiments and analyses were performed separately. Each odor‐stimulation experiment lasted ≈6 min, during which grayscale fluorescence images of mixed hydrogel particles were acquired at 1 fps for ≈300 s (odor sample added at *t* = 30–40 s). Analysis of each dataset (≈240 post‐stimulation frames) required ≈6 min: ≈4.7 min for segmentation, ≈1.3 min for tracking, and ≈8 s for data processing, visualization, and statistical analysis. While the duration of the experimental part is fundamentally limited by the response time of odorant sensor cells, the analysis time could be reduced substantially by lowering the number of processed frames, for example analyzing every 5 s, which would reduce segmentation time to ≈1 min.

Overall, this study establishes a practical framework for the simultaneous analysis of multiple odorant sensor types by combining shape‐encoded hydrogel particles with CNN‐based image analysis. Building on this foundation, future efforts should focus on improving the uniformity of particle fabrication, optimizing odorant delivery protocols, and incorporating higher‐resolution imaging to enhance data quality and strengthen the robustness of the shape‐based analytical platform. Expanding the encoding capacity, either by increasing the number of distinct shapes or integrating hybrid strategies such as color or graphical codes, could enable the detection of a wider range of odorants, taking advantage of the flexibility of the proposed pattern‐based approach. To move toward real‐world implementation, key advances will also be needed: 1) development of a compact measurement device, 2) creation of a complete software pipeline from data acquisition to analysis, 3) identification of relevant odorants and their corresponding odorant receptors, and 4) design of an efficient mechanism to capture airborne odorants into solution. Together, these efforts will help translate this platform into scalable, portable biohybrid odorant sensors for safety, environmental monitoring, and diagnostic use.

## 4 Experimental Section

### Particle Shape‐Based Response Analysis for Odorant Sensor Cells

The particle shape‐based response analysis illustrated in Figure 1 consists of five main steps. First, odorant sensor cells expressing OR, Orco, and the calcium indicator GCaMP were embedded in hydrogel particles, with each cell type embedded in a distinct particle shape. Second, mixtures of the shape‐encoded sensor particles were prepared in a chamber, and odorant samples were added. Third, fluorescence images were acquired over time, beginning from the point of odorant addition (*t* = 0) and continuing throughout the measurement period. Fourth, the particle shapes were automatically identified from the images using a trained image analysis workflow for shape identification. Finally, fluorescence signals from individual particles were categorized by shape and analyzed to evaluate the odorant responses of the corresponding sensor cell types.

### Formation of Hydrogel Particles Containing Odorant Sensor Cells

Odorant sensor cells were ExpiSf9 cells (Gibco) stably expressing ORs, Orcos, and the calcium indicator GCaMP (jGCaMP7^[^ ^43^ ^]^). The cells were kindly provided by Sumitomo Chemical Corporation and cultured in ExpiSf9 CD medium (Gibco) supplemented with 1 × penicillin‐streptomycin (FUJIFILM Wako Pure Chemical Corporation) at 27 °C by shaking at 125 rpm. Cells were passaged every 3–4 days by diluting the culture to ≈1 × 10^6^ cells mL^−1^. When the culture reached a density of ≈1 × 10^7^ cells mL^−1^, cells were harvested by centrifugation (300×*g*, 5 min, room temperature), and the supernatant was gently removed by pipetting.

To prepare agarose‐based particles, 2% (w v^−1^) low‐melting point agarose (Sigma‐Aldrich, A2576) dissolved in 1× HBSS (Hank's Balanced Salt Solution containing calcium, magnesium, without phenol red; hereafter referred to as HBSS(+); Gibco) supplemented with 20 mM PIPES‐NaOH (pH 6.4) was melted in a microwave oven and maintained at 30 °C. The choice of this agarose concentration was based on empirical observation. At 1%, the cell‐containing agarose was fragile (Figure S3, Supporting Information), which made the punching process slightly difficult. It was also considered that higher agarose concentrations potentially reduced odorant diffusivity through the gel pores. Therefore, it was concluded that 2% was optimal. The melted agarose solution was added to the cell pellet and mixed gently by pipetting to achieve a final cell density of ≈1 × 10^8^ cells mL^−1^. The agarose‐cell mixture was then poured between two silicone spacers (thickness: 0.5 mm) adhered to the bottom of a disposable Petri dish or stainless‐steel tray. A glass slide was used to spread the mixture evenly to the spacer‐defined thickness. The dish was transferred to a 4 °C refrigerator for 30 min to allow complete gelation.

To prepare calcium alginate‐based particles, 3% (w v^−1^) sodium alginate dissolved in 1× HBSS (custom‐prepared, without calcium, magnesium, or phenol red) supplemented with 20 mM PIPES‐NaOH (pH 6.4) was added to the cell pellet and mixed by pipetting to reach the same final cell density (≈1 × 10^8^ cells mL^−1^). The alginate‐cell suspension was poured between silicone spacers (thickness: 0.5 mm) fixed to the bottom of a Petri dish or stainless‐steel tray. To initiate gelation, a 100 mM calcium chloride solution in 1 × HBSS(+) supplemented with 20 mM PIPES‐NaOH (pH 6.4) was gently applied over the mixture using a pipette. The mixture was evenly spread to the spacer‐defined thickness using a glass slide. Gelation occurred immediately upon contact with calcium ions at room temperature.

After gelation, the hydrogel sheets were returned to room temperature. Particles were manually excised from the sheet using biopsy punches with reshaped tips (KAI MEDICAL, inner diameter: 2 mm; Figure 2B). During this process, 1× HBSS(+) supplemented with 20 mM PIPES‐NaOH (pH 6.4) was periodically added to the gel surface to prevent dehydration.

### Architecture and Training of CNN Models

A CNN was constructed to classify hydrogel particle shapes into three categories: circle, triangle, and square. The model was implemented using the *Keras* library with a sequential design composed of convolutional, pooling, and dense layers. Grayscale input images were reshaped to 128 × 128 × 1. The architecture began with a convolutional layer using 16 filters (3 × 3 kernel, ReLU activation function), followed by 2 × 2 max pooling. A second convolutional layer with 32 filters (3 × 3, ReLU) and an identical pooling layer was then applied. The resulting feature maps were flattened and passed through a fully connected layer with 64 neurons (ReLU), followed by an output layer with three units corresponding to the shape classes. A softmax function was used to calculate shape probabilities.

The model was compiled using the Adam optimizer, with categorical cross‐entropy as the loss function and classification accuracy as the performance metric. Training was carried out for 20 epochs with a batch size of 128. Five independent models were trained using an 80:20 training‐validation split, and the model with the lowest validation loss was retained in each case. Confusion matrices for training and validation predictions, along with other training metrics, were saved to evaluate training efficiency.

### Preparation of Particle Image Database

To construct a training dataset for shape classification, three sets of sensor particles were prepared with distinct geometries: circle, triangle, and square. Grayscale fluorescence images of particle mixtures containing odorant sensor cells were acquired using an electrophoresis gel imager by detecting GCaMP fluorescence (Figure 2D (i)). Image segmentation was performed using *Cellpose 2.0* ^[^ ^23^, ^24^ ^]^ (Figure 2D (ii)), yielding binary masks in which background pixels were assigned a value of 0 and particle regions a value of 1. To exclude broken or aggregated particles, lower and upper size thresholds (2000 and 3400 pixels, respectively) were applied. While this filtering reduced contamination by broken, overlapping, or poorly segmented particles, it did not eliminate them entirely. Each valid particle mask was assigned a unique index, and the corresponding grayscale region was extracted from the original image using Boolean masking (Figure 2D (iii)). The extracted grayscale particles were centered in 128 × 128 pixel zero‐padded images. The resulting individual particle images were then visually inspected and manually sorted into shape‐specific folders, yielding ≈2600 images per shape (circle, triangle, and square; Figure 2D (iv)).

### Design of Particle Shape Identification Scheme

A particle shape identification scheme was developed to classify sensor particles in new test images using five independently trained CNN classifiers (Figure 2H). Grayscale fluorescence images were again segmented using *Cellpose 2.0*,^[^ ^23^, ^24^ ^]^ and individual particle images were extracted and centered within 128 × 128 pixel zero‐padded images, as described above. These standardized particle images were input into the five trained CNN models, each returning a softmax probability vector representing classification confidence across shape categories. The five vectors were averaged to produce a single probability vector. If the maximum probability in this averaged vector exceeded a threshold of 0.5, the corresponding shape was assigned as the predicted label; otherwise, the particle was labeled as “unidentified.” This process was referred as a shape “identification” scheme rather than a classification scheme, as it incorporates post‐classification steps such as softmax averaging, confidence thresholding, and the introduction of a fourth category for uncertain predictions. This ensemble‐based approach enabled robust and automatic identification of particle shapes under conditions not used during training.

### Single Odorant Addition Experiment

The three types of agarose particles were, each containing odorant sensor cells expressing a different OR along with the same Orco and GCaMP. The three ORs (AgOR1,^[^ ^25^ ^]^ AaOR4,^[^ ^26^ ^]^ and AaOR15^[^ ^27^ ^]^) were selected based on their reported responsiveness to specific odorants: phenol for AgOR1, 6‐methyl‐5‐hepten‐2‐one for AaOR4, and acetophenone for AaOR15. Each sensor cell type was embedded in agarose particles of a distinct shape: circle for AgOR1‐expressing cells, triangle for AaOR4, and square for AaOR15 (Figure 3A).

The shaped particles were mixed in 1× HBSS(+) supplemented with 20 mM PIPES‐NaOH (pH 6.4). Odorant stock solutions were prepared by dissolving each compound (phenol, 6‐methyl‐5‐hepten‐2‐one, or acetophenone) at 100 mM in DMSO, then diluting them in 1 × HBSS(+) supplemented with 20 mM PIPES‐NaOH (pH 6.4) to a working concentration of 0.25 mM (DMSO content, 0.25% v v^−1^).

At the start of each experiment, blue excitation light was turned on, and time‐lapse fluorescence imaging was initiated. Images were acquired at 1 fps for ≈5 min and continued throughout the observation period. After ≈30 s, 1000 µL of odorant solution (0.25 mM) was added to the particle mixture (initial volume: 1.5 mL) by dividing it into six 167 µL portions and sequentially adding them at six different locations (Figure S5, Supporting Information). This resulted in a final odorant concentration of 0.1 mM and 0.1% v v^−1^ DMSO, corresponding to a 2.5‐fold dilution. 0.1 mM was chosen for the experiment based on dose‐dependent responses of the same cells reported in a separate study on 2D particle arrays.^[^ ^42^ ^]^

### Shape‐Based Fluorescence Analysis of Odorant Sensor Particles

The shape identification scheme was applied to each frame of the time‐lapse fluorescence images obtained from the odorant addition experiments, enabling particles with unique indices to be identified across all frames. To follow the motion of slightly jiggling particles over time, pairwise distances were calculated between particle centroids in consecutive frames (1 s intervals) and linked them based on spatial proximity. Tracking was performed from the frame at which odorant addition was completed (*t* = 0 min) to the final frame of the measurement (*t* = 4 min). Once particle trajectories were established, the mean fluorescence intensity of each particle (*F*(*t*)) was computed in every frame, and intensity profiles were grouped by particle shape and plotted over time.

### Statistical Analysis

Multiple comparisons in Figure 3D–G(iv) were performed as follows. First, a Kruskal‐Wallis test was used to assess overall differences among groups. Subsequently, Dunn's *post‐hoc* test with Bonferroni correction was applied for pairwise comparisons. All analyses were conducted using the *scipy.stats* and *scikit_posthocs* packages in Python. All *p*‐values were rounded to the first significant digit and annotated using the conventional method: “n.s.” (not significant) for *p* > 0.05, “*” for *p* ≤ 0.05, “**” for *p* ≤ 0.01, and “***” for *p* ≤ 0.001. The results of the statistical analyses are summarized in Tables S1–S8 (Supporting Information).

## Conflict of Interest

The authors declare no conflict of interest.

## Author Contributions

S. Takamori, H.M., T.K., T. Osaki, and N.M. performed the conceptualization. S. Takamori, T. Ohnishi, and T.K. performed the investigation. S. Takamori performed the visualization. H.M., T. Osaki, N.M., and S. Takeuchi performed the supervision. S. Takamori wrote the original draft. S. Takamori, T.K., T. Ohnishi, H.M., T. Osaki, N.M., and S. Takeuchi performed the writing, reviewing and editing.

## Supporting information



Supporting Information

## Data Availability

The data that support the findings of this study are available from the corresponding author upon reasonable request.
