# Supplementary material for: Shape‐Encoded Hydrogel Sensor Particles Enable Multiplex Odorant Detection Through Deep‐learning Classification
Source: Small. 2025 Oct 22;21(49):e07903. doi: 10.1002/smll.202507903 (PMC12696800; doi:10.1002/smll.202507903)
Supplement: Supplementary file 1 — Supporting Information [file SMLL-21-e07903-s001.docx]

Supporting Information for

**Shape-encoded hydrogel sensor particles enable multiplex odorant detection through deep-learning classification**

Sho Takamori,^[a]^ Taisei Kawakami,^[a,b]^ Tomoko Ohnishi,^[a]^ Hisatoshi Mimura,^[a]^ Toshihisa Osaki,^[a]^ Norihisa Miki,^[a,b]^ and Shoji Takeuchi*^[a,c,d]^

1. Dr. S. Takamori, Mr. T. Kawakami, Ms. T. Ohnishi, Dr. H. Mimura, Dr. T. Osaki, Prof. Dr. N. Miki, Prof. Dr. S. Takeuchi*

Artificial Cell Membrane Systems Group, Kanagawa Institute of Industrial Science and Technology, 3-2-1 Sakado, Takatsu-ku, Kawasaki, Kanagawa 213-0012, Japan.

Email: takeuchi@hybrid.t.u-tokyo.ac.jp

1. Mr. T. Kawakami, Prof. Dr. N. Miki

Department of Mechanical Engineering, Faculty of Science and Technology, Keio University, 3-14-1 Hiyoshi, Kohoku-ku, Yokohama, Kanagawa 223-8522, Japan.

1. Prof. Dr. S. Takeuchi*

Institute of Industrial Science, The University of Tokyo, 4-6-1 Komaba, Meguro-ku, Tokyo 153-8505, Japan.

1. Prof. Dr. S. Takeuchi*

Department of Mechano-Informatics, Graduate School of Information Science and Technology, The University of Tokyo, 7-3-1 Hongo, Bunkyo-ku, Tokyo 113-8656, Japan.

**Supplementary Figures**


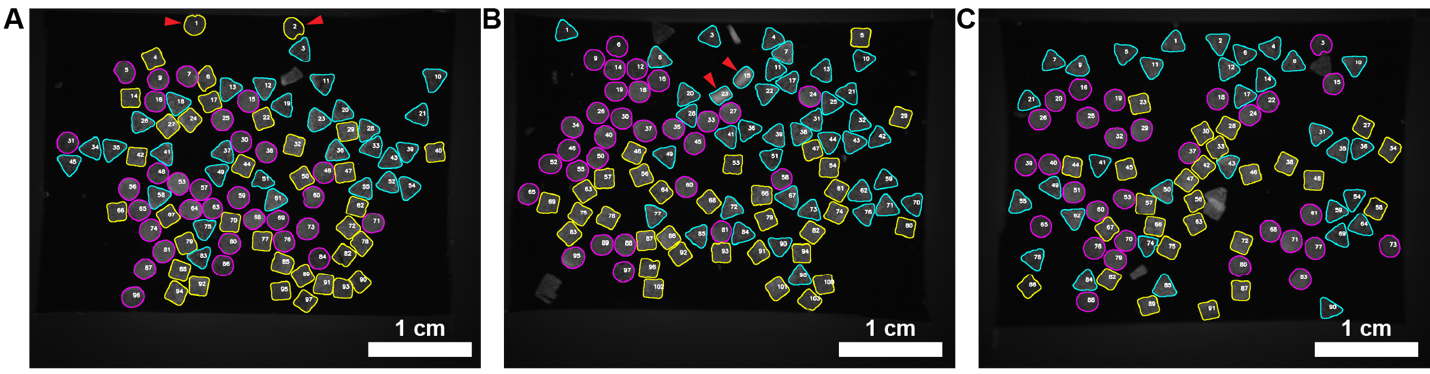


**Figure S1. Results of particle shape identification.** Odorant added: phenol (panel A), 6-methyl-5-hepten-2-one (panel B), none (DMSO; panel C). The numbers on the particles indicate individual unique index for identification. Red arrowheads indicate particles with shape misidentification.

**Figure S2. Variability in fluorescence responses among individual odorant sensor cells.** Sensor cells expressing AgOR15 were exposed to 0.1 mM acetophenone at *t* = ~10 s. (A) Fluorescence micrographs before and after odorant addition. Cells with no apparent overlap were manually segmented; segmentation boundaries are highlighted in red. (B) Plots of mean fluorescence intensity over time for the segmented cells shown in (A).


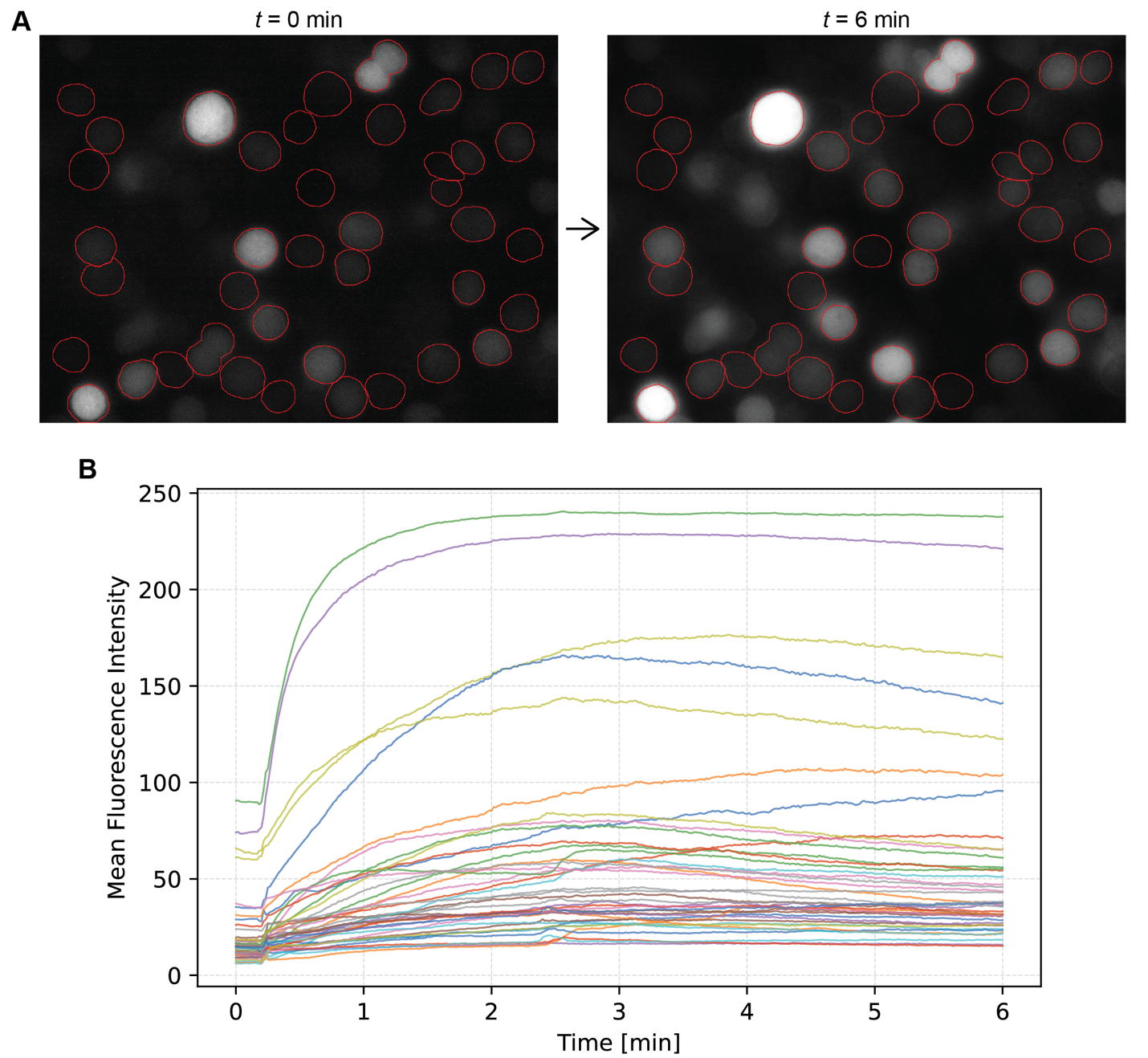


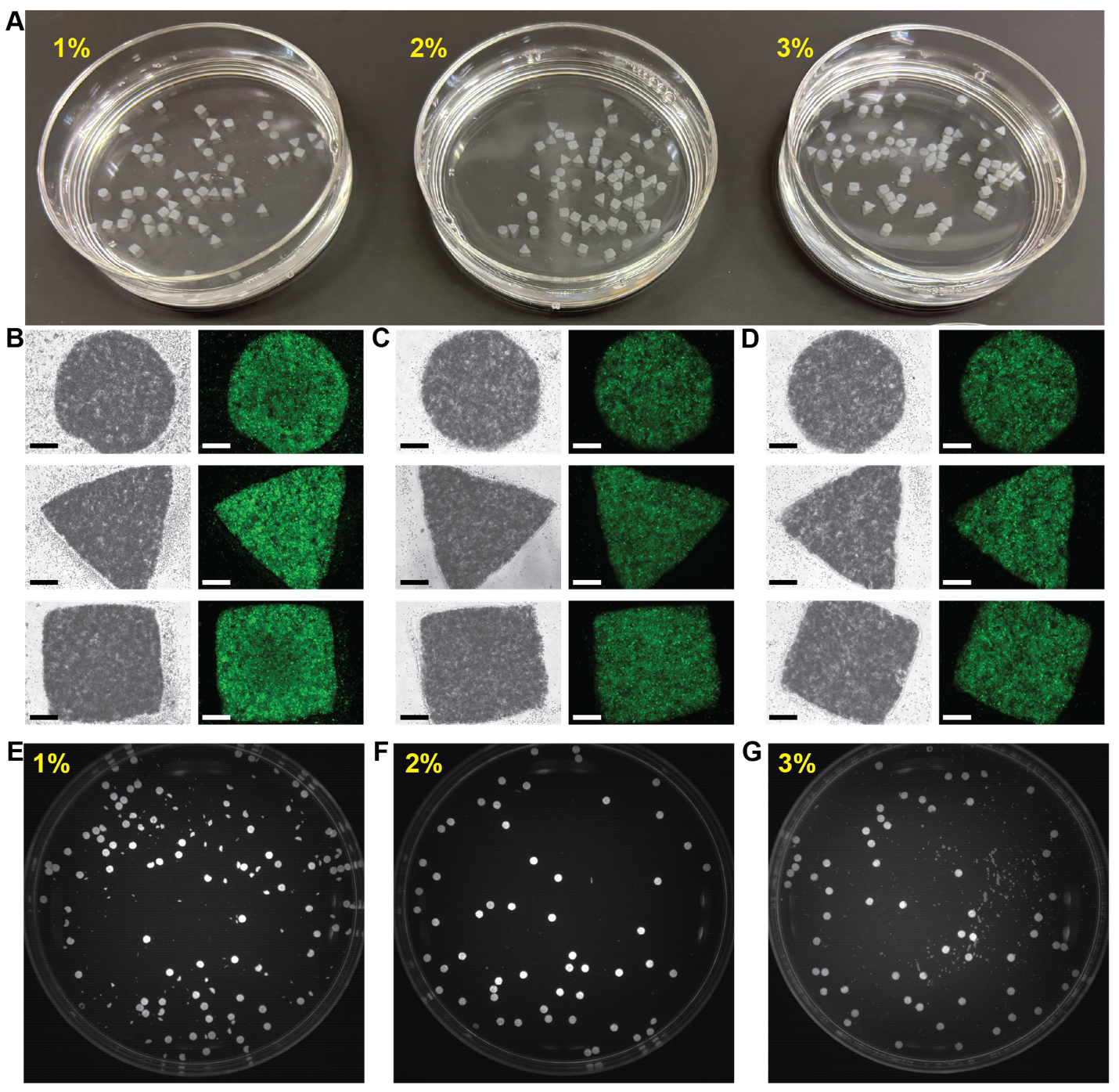


**Figure S3. Cell-containing particles at different concentrations of agarose.** (A) Particles prepared at 1%, 2%, 3% agarose in 1× HBSS(+) with 20 mM PIPES-NaOH (pH 6.4). (B) Brightfield and green fluorescence micrograph of representative particles at 1%. (C) Brightfield and green fluorescence micrograph of representative particles at 2%. (D) Brightfield and green fluorescence micrograph of representative particles at 3%. (E) 1% agarose particles (circle only) containing cells after 10 s of vortexing at 3,000 rpm. (F) 2% agarose particles (circle only) containing cells after 10 s vortexing at 3,000 rpm. (E) 3% agarose particles (circle only) containing cells after 10 s vortexing at 3,000 rpm. Scale bar: 500 μm.


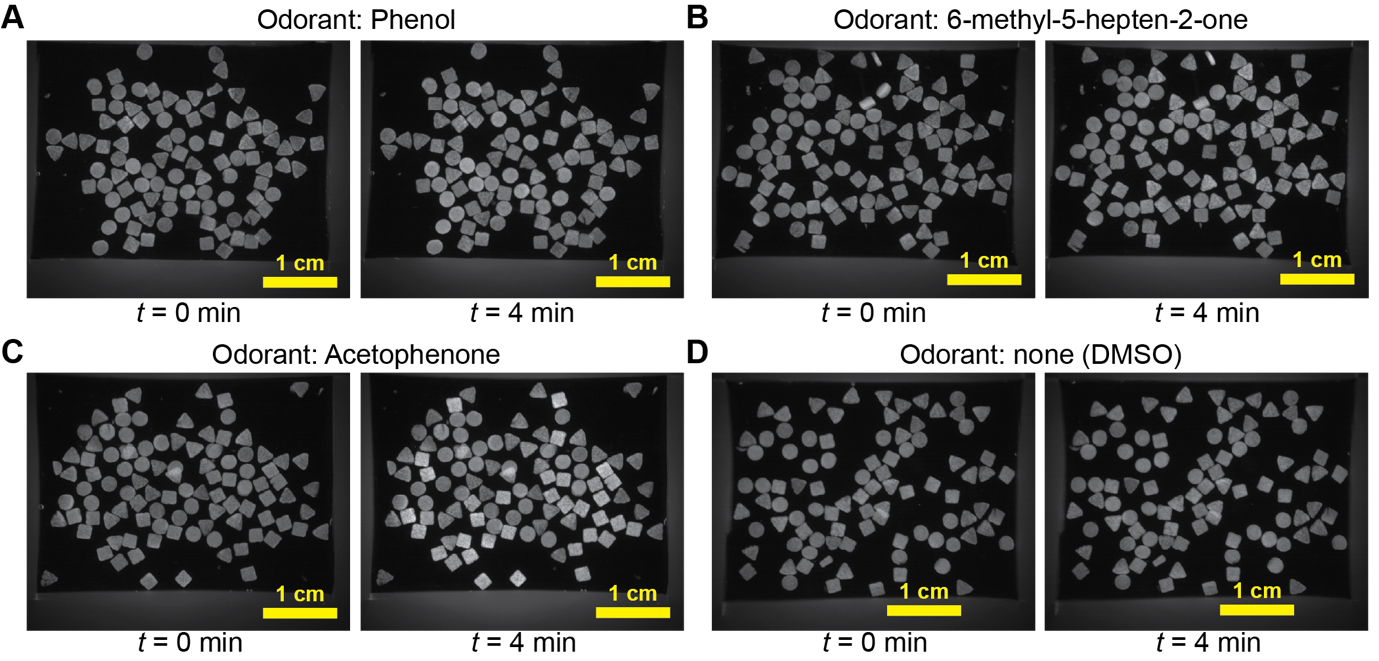


**Figure S4. Grayscale hydrogel fluorescence images before (*t* = 0 min) and after (*t* = 4 min) the addition of corresponding odorant.** (A) Odorant: phenol. (B) Odorant: 6-methyl-5-hepten-2-one. (C) Odorant: Acetophenone. (D) Odorant: none (DMSO).


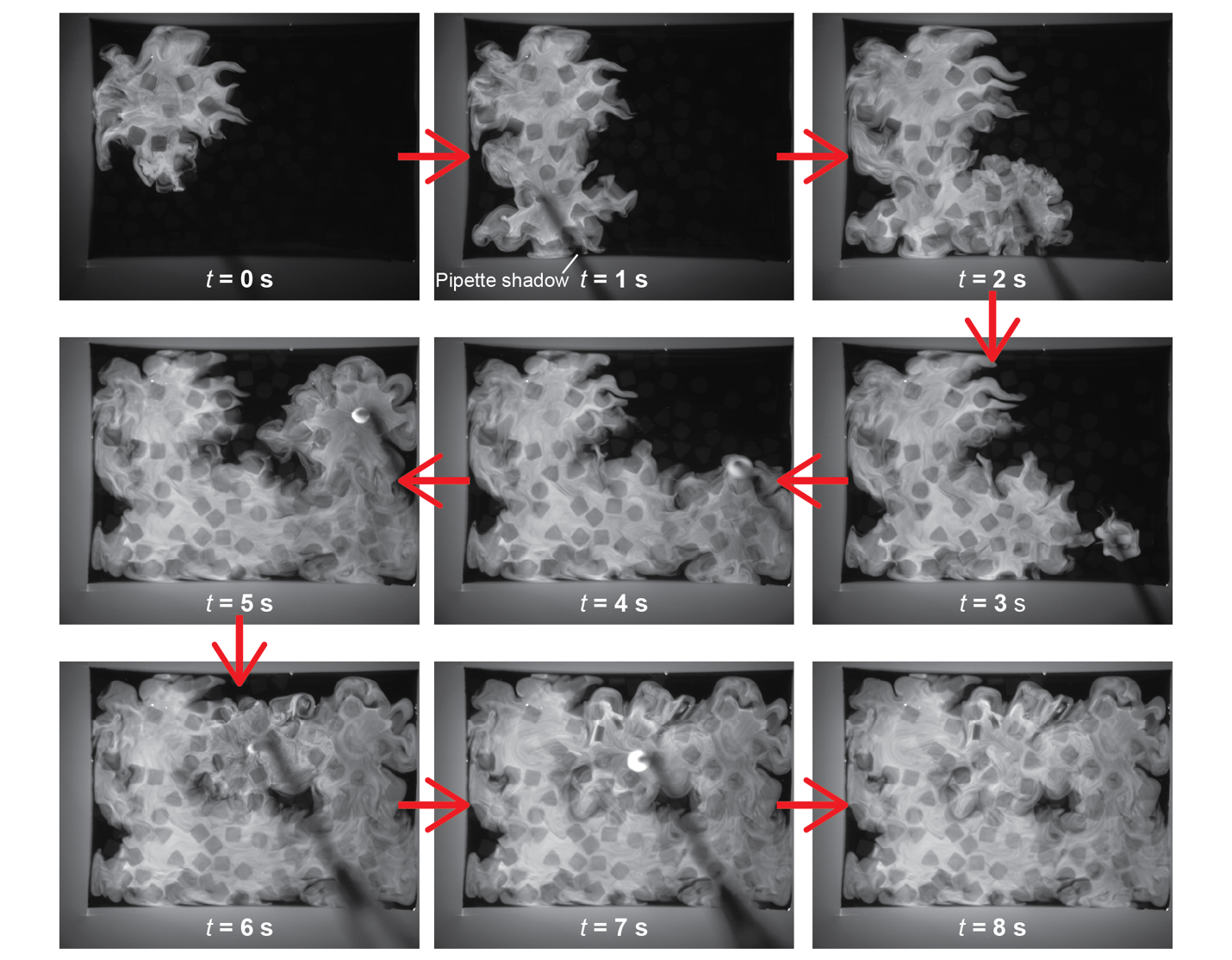


**Figure S5. Sample deposition test using fluorescent dye.** Calcein solution was added to an ~3 × 4 cm^2^ well by dividing the total volume into six aliquots and dispensing them sequentially at different positions within the well. As shown, the full deposition process typically required approximately 7 s.

**
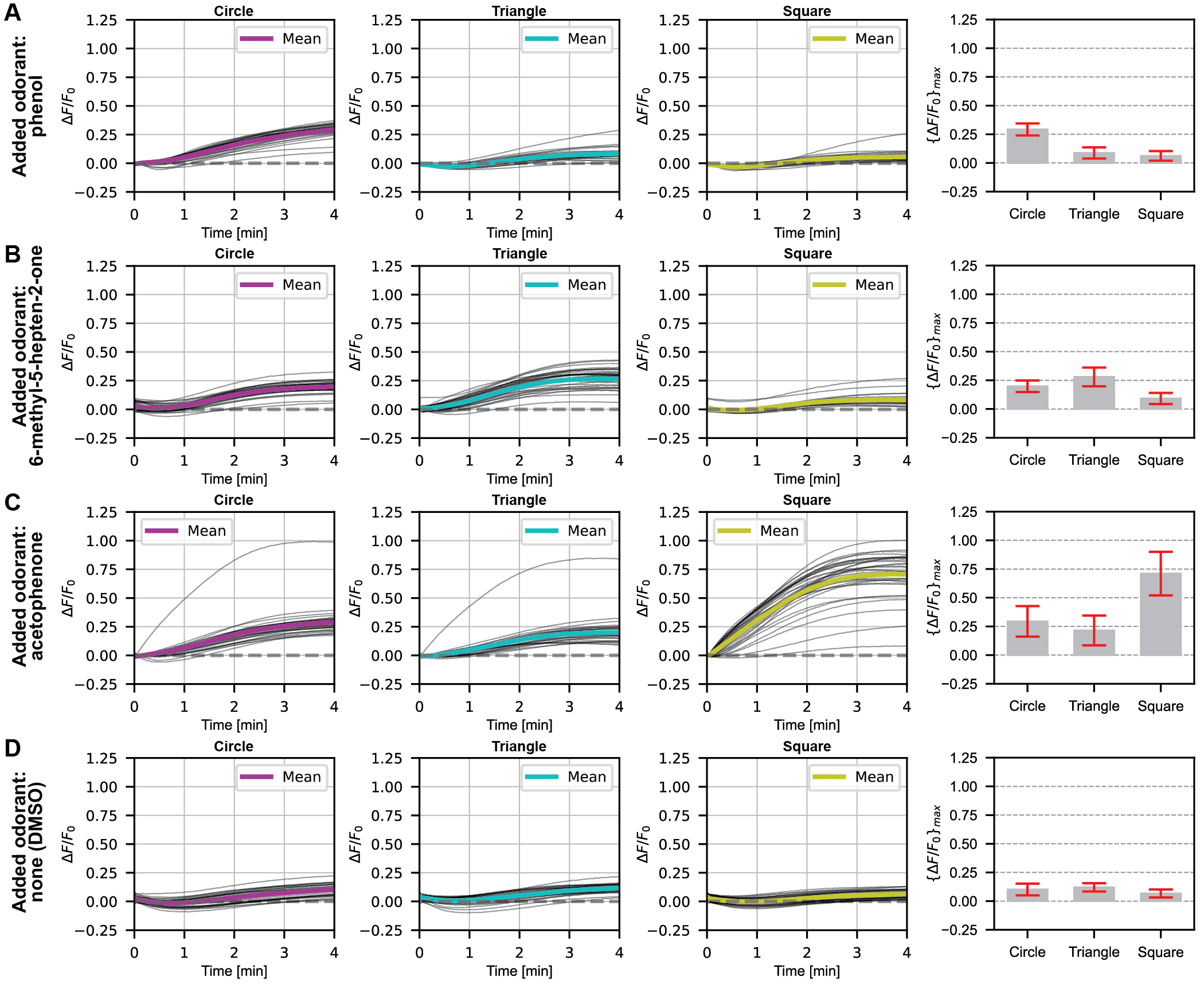
**

**Figure S6. Shape-based analysis of fluorescence responses in odorant sensor particles.** The odorant stimulation experiment shown in Fig. 3 of the main text was repeated on a different day. The experimental procedure was the same as described in Section 4 (Experimental Section, Single odorant addition experiment) of the main text. Fluorescence intensity changes in identified particles and their maximum values during the 4 min observation are plotted, indicating reproducible responsiveness to single-odorant stimulation.

**
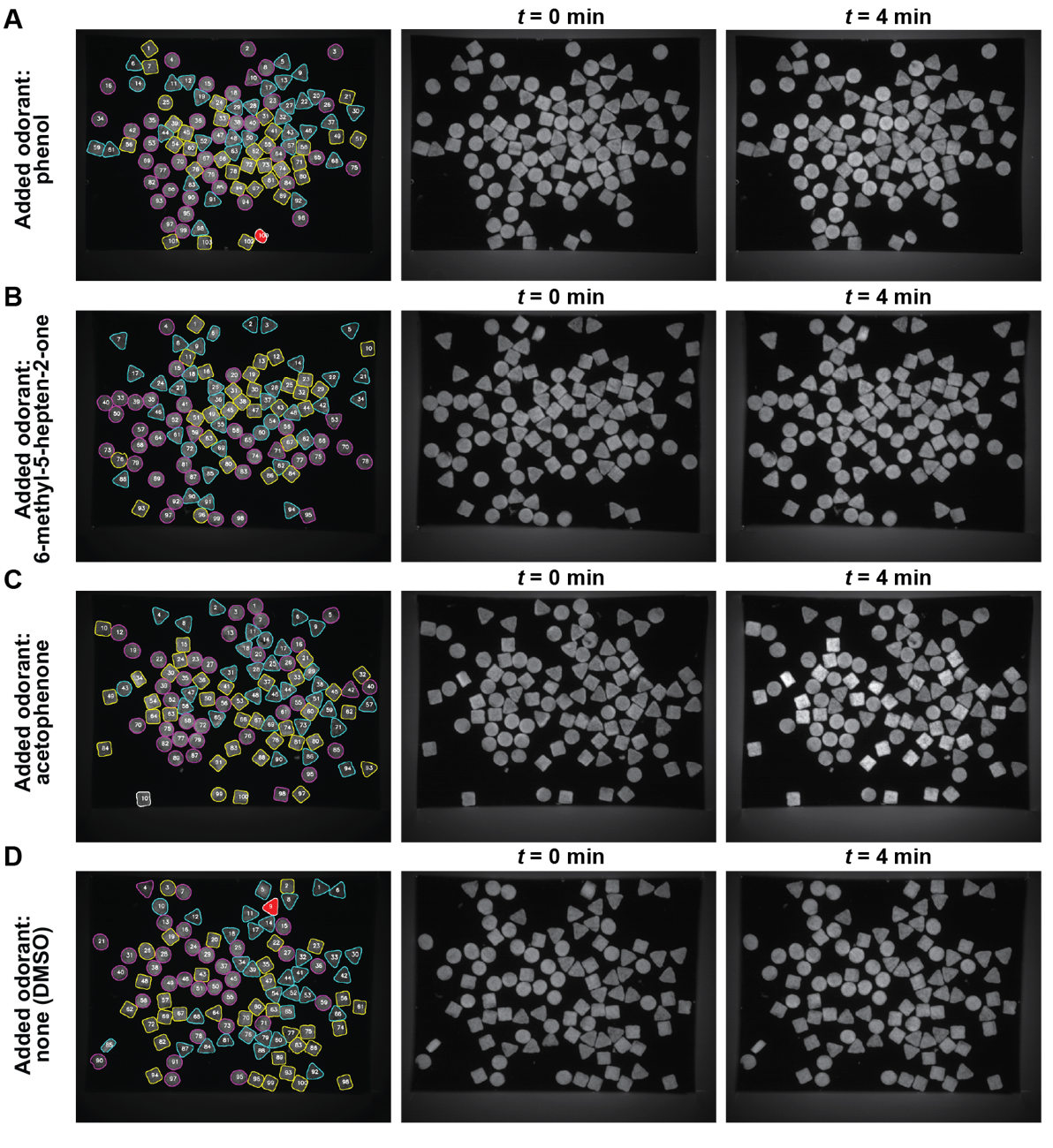
**

**Figure S7. Shape identification and fluorescence response of odorant sensor particles.** The odorant stimulation experiment shown in Fig. 3 of the main text was repeated on a different day. The experimental procedure was the same as described in Section 4 (Experimental Section*,* Single odorant addition experiment) of the main text. Results of shape identification and images before (*t* = 0 min) and after (*t* = 4 min) odorant addition are shown in the left, middle, and right panels, respectively.

**Supplementary Tables**

| Comparison Group | Test Statistic (H) | Degrees of Freedom | *p*-value |
| --- | --- | --- | --- |
| Circle vs Triangle vs Square | 56.5 | 2 | 5.3E-13 |

**Table S1. Results of Kruskal-Wallis test for the shape-based fluorescence response analysis shown in Fig. 3D (odorant: phenol).**

| Group 1 | Group 2 | Adjusted *p*-value (Bonferroni) | Significant (*α* = 0.05) |
| --- | --- | --- | --- |
| Circle | Square | 2.5E-12 | Yes |
| Circle | Triangle | 5.1E-08 | Yes |
| Square | Triangle | 0.59 | No |

**Table S2. Results of Dunn’s *post-hoc* test with Bonferroni correction for the shape-based fluorescence response analysis shown in Fig. 3D (odorant: phenol).**

| Comparison Group | Test Statistic (H) | Degrees of Freedom | *p*-value |
| --- | --- | --- | --- |
| Circle vs Triangle vs Square | 72.8 | 2 | 1.6E-16 |

**Table S3. Results of Kruskal-Wallis test for data shown in Fig. 3E (odorant: 6-methyl-5-hepten-2-one).**

| Group 1 | Group 2 | Adjusted *p*-value (Bonferroni) | Significant (*α* = 0.05) |
| --- | --- | --- | --- |
| Circle | Square | 0.00049 | Yes |
| Circle | Triangle | 5.0E-05 | Yes |
| Square | Triangle | 5.6E-17 | Yes |

**Table S4. Results of Dunn’s *post-hoc* test with Bonferroni correction for data shown in Fig. 3E (odorant: 6-methyl-5-hepten-2-one).**

| Comparison Group | Test Statistic (H) | Degrees of Freedom | *p*-value |
| --- | --- | --- | --- |
| Circle vs Triangle vs Square | 70.2 | 2 | 5.7E-16 |

**Table S5. Results of Kruskal-Wallis test for data shown in Fig. 3F (odorant: acetophenone).**

| Group 1 | Group 2 | Adjusted *p*-value (Bonferroni) | Significant (*α* = 0.05) |
| --- | --- | --- | --- |
| Circle | Square | 2.4E-07 | Yes |
| Circle | Triangle | 0.078 | No |
| Square | Triangle | 1.0E-15 | Yes |

**Table S6. Results of Dunn’s *post-hoc* test with Bonferroni correction for data shown in Fig. 3F (odorant: acetophenone).**

| Comparison Group | Test Statistic (H) | Degrees of Freedom | *p*-value |
| --- | --- | --- | --- |
| Circle vs Triangle vs Square | 50.7 | 2 | 9.7E-12 |

**Table S7. Results of Kruskal-Wallis test for data shown in Fig. 3G (odorant: none).**

| Group 1 | Group 2 | Adjusted *p*-value (Bonferroni) | Significant (*α* = 0.05) |
| --- | --- | --- | --- |
| Circle | Square | 2.8E-10 | Yes |
| Circle | Triangle | 1 | No |
| Square | Triangle | 5.7E-09 | Yes |

**Table S8. Results of Dunn’s *post-hoc* test with Bonferroni correction for data shown in Fig. 3G (odorant: none).**
